# High Concussion Rate in Student Community Rugby Union Players During the 2018 Season: Implications for Future Research Directions

**DOI:** 10.3389/fnhum.2019.00423

**Published:** 2019-12-04

**Authors:** James Craig Brown, Lindsay Toyah Starling, Keith Stokes, Pierre Viviers, Esme Jordaan, Sean Surmon, Elton Wayne Derman

**Affiliations:** ^1^Department of Surgical Sciences, Division of Orthopaedics, Institute of Sport and Exercise Medicine, Stellenbosch University, Cape Town, South Africa; ^2^IOC Research Centre, Cape Town, South Africa; ^3^Department for Health, University of Bath, Bath, United Kingdom; ^4^Rugby Football Union, Twickenham, United Kingdom; ^5^Biostatistics Unit, Medical Research Council, Cape Town, South Africa; ^6^Statistics and Population Studies Department, University of the Western Cape, Cape Town, South Africa; ^7^Maties Sport, Stellenbosch University, Stellenbosch, South Africa

**Keywords:** non-professional, injury surveillance, football, Stellenbosch, video

## Abstract

Collision sports, such as Rugby Union (“Rugby”) have a particularly high risk of injury. Of all injuries common to collision sports, concussions have received the most attention due to the potentially negative cognitive effects in the short- and long-term. Despite non-professional Rugby players comprising the majority of the world’s playing population, there is relatively little research in this population. Stellenbosch Rugby Football Club (“Maties”), the official rugby club of Stellenbosch University, represents one of the world’s largest non-professional Rugby clubs, making this an ideal cohort for community-level injury surveillance. The aim of this study was to describe the incidence and events associated with concussion in this cohort. Baseline demographics were obtained on the 807 male student Rugby non-professional players who registered for the 10-week long 2018 season, which comprised 101 matches and 2,915 of exposure hours. All match-related injuries were captured by the medical staff of Stellenbosch Campus Health Service on an electronic form developed from the consensus statement for injury recording in Rugby. The mean age, height and weight of this cohort were 20 ± 2 years, 182 ± 7 cm and 88 ± 14 kg, respectively. Overall, there were 89 time-loss injuries, which equated to an injury rate of 30.6 per 1,000 match hours [95% confidence intervals (CIs): 24.2–36.9], or about one injury per match. The most common injury diagnosis was “concussion” (*n* = 27 out of 90 injuries, 30%), at a rate of 9.3 per 1,000 match hours (95% CIs: 5.8–12.8). The three most common mechanisms of concussion in the present study were performing a tackle (33%), accidental collision (30%) and being tackled (11%). Concussion was the most common injury in this population, at a rate that was six times higher than the most comparable study from the UK, which had far more exposure time over six seasons and wider range of player ability, from recreational to semi-professional. This might be explained by the training and vigilance of the club’s first aiders observing all matches for concussion. Future studies should try to explain this high rate and subsequently reduce these concussions. The addition of video surveillance data would assist in identifying the etiology of these concussions injuries in order to develop specific targeted interventions.

## Introduction

Despite the many benefits of participating in sports such as Rugby Union (“Rugby”), this activity also carries a higher risk of injury to players than many other popular sports (Allender et al., 2006; Williams et al., 2013). Moreover, Rugby is also associated with head injuries and concussion (Williams et al., 2013; Roberts et al., 2017). Sports-related concussions have received substantial attention in empirical and lay literature, mainly due to concussion’s proposed link with long-term cognitive impairment (Wiebe et al., 2011; Smith et al., 2019).

The majority of the World’s Rugby playing population is non-professional (World Rugby, 2018) but there are relatively few injury surveillance studies in this cohort (Viviers et al., 2018; Yeomans et al., 2018). A meta-analysis of the six studies at the non-professional level (Yeomans et al., 2018) concluded that the match injury rate was 46.8/1,000 player hours [95% confidence interval (CI) 34.4–59.2], which is significantly lower than the rate for the professional level of the game at 81 per 1,000 player hours (95% CI 63–105; Williams et al., 2013). However, in a study of college students in the United States with a similar injury definition (Kerr et al., 2008), the match injury rate was substantially lower at 16.9 per 1,000 match hours (95% CI: 15.1–18.9); and in a three-season UK study of non-professional adult players, the match injury rate, for injuries resulting in more than 7 days’ time-loss, was 14.2 injuries per 1,000 h (95% CIs: 13.0–15.5; Roberts et al., 2013).

Stellenbosch Rugby Football Club (SRFC) is one of the largest Rugby non-professional clubs in the world, with about 1,000 registered players. The club size makes it a representative sample of the non-professional playing population. Based on a study conducted in a similarly non-professional cohort in the United Kingdom (UK; Roberts et al., 2017), these koshuis players could be described as “recreational,” with a few aspirational “semi-professional” players. The meta-analysis of amateur rugby studies reported that knee, shoulder, thigh, ankle, neck and back areas were commonly injured regions (Yeomans et al., 2018). An injury surveillance study conducted in SRFC koshuis players comparing 1973–1975 with 2003–2005 found that the proportion of head/face injuries had increased over time (Puren et al., 2007). Other commonly injured regions in this cohort were the knee, ankle and shoulder. This concurs with the common injury areas of student rugby players of the United States (Kerr et al., 2008).

As one might expect with the high proportion of head injuries, a tackle injury epidemiology study in the SRFC koshuis cohort between 2012 and 2013 found a concussion incidence rate (IR) of 1.4 per 1,000 match hours (95% CIs: 1.2–1.6; Freitag et al., 2015). This rate is similar to those of non-professional cohorts in the United Kingdom (UK) between 2009 and 2015 (Roberts et al., 2017) and collegiate cohort in the United States of America (USA) which reported concussion rates of 1.6 (95%CI: 1.2–1.9) and 2.2 (95% CI 1.5–2.8) per 1,000 match hours, respectively (Kerr et al., 2008). This also falls within the range reported for semi-professional and community level male players in a meta-analysis of concussions rates: average: 2.1 per 1,000 player hours (range: 1.2–6.9), although this includes training-related injury rates (Gardner et al., 2014). By contrast, rugby league—a variant of rugby union with more emphasis on the tackling phase of play—(Freitag et al., 2015) reported significantly higher match-related concussion rates in amateur players (19.1 per 1,000 match hours, 95% CIs: 14.5–25.3).

However, the previous study in SRFC residence players only focused on injuries resulting from the tackle phase of play, which might underestimate the total number of concussions. Moreover, evidence from professional Rugby suggests that concussion reporting has improved exponentially since the years when the SRFC, UK and USA studies were conducted (Project Steering Group, 2018). Also, despite numerous concussion education programmes, there is little evidence of their effectiveness to date (Fraas and Burchiel, 2016).

Thus, the aim of this study was to describe the contemporary concussion injury rate and factors (body region, phase of play, level of play, et cetera) associated with these injuries in this large cohort of non-professional student rugby players at SRFC.

## Materials and Methods

The participants were all registered players of the residence (“koshuis”) rugby tournament at Stellenbosch University that usually includes about 200 matches over 7 months. Except for one “private-residence” team, all teams comprise Stellenbosch University students. Baseline details were collected on all of these registered Rugby players including self-reported weight, height and age. An injury surveillance form was developed for the university’s medical staff based on the consensus statement for injury reporting in Rugby Union (Fuller et al., 2007). These staff included sports physicians, clinical nurse practitioners, physiotherapists and first aiders. Educational training of medical staff was conducted on the use of the form prior to its implementation. Ethics approval to conduct this study was granted by Stellenbosch University (HREC B17/10/003).

The medical staff, including first aiders, all complete World Rugby concussion training^1^. Each match is assigned a first aider in order to observe and identify suspected concussions based on World Rugby “recognize and remove guidelines” signs: convulsive, headache, “knocked out,” nauseous, unsteady, confused, dazed, dizzy^1^. If this, or any other sign/symptom is observed by a first aid personnel, they are instructed to remove the player(s) immediately and take them to the medical room for assessment. These players are considered concussed under the “recognize and remove” protocol and are unable to return to that match (Echemendia et al., 2017). All other injuries were reported voluntarily by players to the medical staff either present on each night of matches or at the university medical clinic in the days following a match. Although seeking medical assistance at the university medical clinic incurs a fee for patients, this fee is subsidized by the university and thus probably cheaper than any other private health care provider in the area. This subsidization would reduce the likelihood of missed injuries.

However, although all medical attention (those seeking medical attention) were captured, we will only report on time-loss injuries in this study, which according to the consensus statement: *“…results in a player being unable to take a full part in future rugby training or match play*” (Fuller et al., 2007).

In addition to all injuries being captured, the total number of matches were recorded from the SRFC administrators. The total match exposure time was calculated, as recommended by the consensus statement for injury reporting (Fuller et al., 2007) by multiplying the number of matches by 30 players by the duration of the match. Total match duration was 60 min for leagues 1–3, and 50 min for 4th league. In this way, injury rates were calculated (described in “Statistical Analyses” section) as the main outcome. Other variables that were recorded on the injury recording form included body region, phase of play/activity that caused the injury, league (1st–4th, 1st is highest and 4th lowest), injury management (referral), method of arrival, diagnosis code: orchard Sports Injury Classification System (OSICS; Rae and Orchard, 2007), and severity (“minimal”: 2–3 days lost; “mild”: 4–7 days; “moderate”: 8–28 days; “severe”: >28 days). Body region was captured by OSICS code but was further categorized into the four regions used in the Williams et al. (2013) meta-analysis for comparison: head/neck, upper body, trunk and lower body.

Overall, 101 matches were played over the 10 weeks of the koshuis tournament. The 1st–3rd league matches were 60 min in length, while the lowest (4th) league matches were 50 min in length. In most of the 10 weeks, the four leagues played on four different nights of the week, starting with fourth league, and ending with first league matches. Assuming 30 players per match, this produced an estimated total exposure time of 2,910 h (Table 1).

**Table 1:** Koshuis 2018 season match count, duration, exposure and number of time-loss injuries and incidence rates, by league.

League	Count of matches	Match duration (min)	Match exposure (h)	Match injury count (*n*)	Overall match injury rate (IR) per 1,000 h (95% CI)
1	40	60	1,200	35	29.2 (19.5–38.8)
2	20	60	600	30	50.0 (32.1–67.9)
3	17	60	510	9	17.6 (6.1–29.2)
4	24	50	600	13	21.7 (9.9–33.4)
*Unknown*	-	-	-	2	1.7 (0–4.0)
**Total**	**101**		**2,910**	**89**	**30.6 (24.2–36.9)**

### Statistical Analyses

The outcome variable was the number of: (1) overall time-loss injuries; and (2) concussions during the matches played in the 2018 season. The measure of risk was expressed as the IR, calculated as the number of injuries/the total number of match exposure hours for the season. Exact 95% CIs were reported (Ulm, 1990; Rothman and Greenland, 1998). The number of injuries and the IR was calculated for various categories of relevant variables based on the rugby injury reporting consensus statement: (Fuller et al., 2007) league (level of play), body region, phase of play/activity that caused the injury.

Injury rates were considered statistically different at the 5% level if their two corresponding 95% CIs did not overlap (Cumming, 2009).

## Results

In total, 807 koshuis players were registered for the 2018 season in four leagues, depending on level of play. The mean age of these players was 20 ± 2 years, and the mean height and weight was 182 ± 7 cm and 88 ± 14 kg, respectively.

During this 2018 koshuis tournament, there were 89 match-related time-loss injuries at mean injury rate of 30.6 per 1,000 match hours (95% CIs: 24.2–36.9; Table 1). On the assumption that each of the 807 players played a match, this is an injury proportion of 11.0% (95% CIs: 9.0–13.4).

Of the four regions (Head/neck, Upper limb, Trunk, Lower limb,) the most frequently injured was the head/neck (Table 2), which comprised almost half of the injuries (44%, *n* = 39 of 89 injuries) in these four regions at a IR of 14.4 (95% CI: 10.1–18.8) per 1,000 match hours.

**Table 2:** Match-related time loss-Injuries by body region; the number, proportion and incidence rate (IR; 95%CI).

Location	Count	Proportion of all injuries	Overall match injury rate (IR) per 1,000 h (95%CI)
Head/Neck	39	44%	14.4 (10.1–18.8)
Upper limb	25	28%	9.3 (5.8–12.8)
Lower limb	25	28%	8.6 (5.2–12.0)
Trunk	0	-	-

*CI, confidence interval*.

In terms of specific regions as identified by the OSICS code, the five most commonly injured regions, which comprised about 90% of all injuries, were the head (44%, *n* = 39), shoulder (18%, *n* = 16), ankle (12%, *n* = 11), knee (9%, *n* = 8) and hand (6%, *n* = 5). The following three OSICS codes were reported by medical staff and grouped together for “concussion”: HNXX (*n* = 9), HNCA (*n* = 13) and HNCX (*n* = 5). Collectively, and irrespective of region, these three concussion codes made concussion was the most common overall diagnosis (*n* = 27, 30% of all diagnoses) at a rate of 9.3 per 1,000 match hours (95% CIs: 5.8–12.8). Twenty-six players sustained these 27 recorded concussions. Other frequently reported OSICS codes were shoulder dislocation (SDAX/SDXX: *n* = 5), AC joint sprain (SJAX, *n* = 3), shoulder subluxations (SJSA/SJXX, *n* = 4) and general shoulder injuries (SXXX, *n* = 2). The most frequently recorded phase of play (Table 3) that was associated with concussions was tackling (33%), followed by accidental collisions that were *not* part of the tackle phase of play (30%). This was slightly different from the ranking of proportions for overall match injuries where tackling (28%) and being tackled (22%) were the two most commonly reported phases of play. In Table 4, overall match and concussion rates were compared by level of play (four leagues). The 2nd league had a significantly higher overall injury rate, in comparison to the 3rd league. The concussions rates were highest for the 2nd and 1st leagues, but the rates were not significantly higher than the 3rd and 4th leagues. Although the third league had the lowest rate, this was not significantly lower than any other league.

**Table 3:** Phases of play associated with overall match injuries (total of 89) and match concussion injuries (total of 27); the number, proportion and IR (95%CI).

	Overall time-loss match injury count (% of all injuries)	Overall time-loss match injury rate (IR) per 1,000 h (95%CI)	Concussion match injury count (n, %)	Concussion match injury rate (IR) per 1,000 h (95%CI)
Overall	89 (100%)	30.6 (24.2–36.9)	27 (100%)	9.3 (5.8–12.8)
Tackling	25 (28%)	8.6 (5.2–12.0)	9 (33%)	3.1 (1.1–5.1)
Tackled (ball-carrier)	20 (22%)	6.9 (3.9–9.9)	3 (11%)	1.0 (0–2.2)
Accidental collision (not part of a tackle)	16 (18%)	5.5 (2.8–8.2)	8 (30%)	2.7 (0.8–4.7)
Non-contact*	10 (11%)	3.4 (1.3–5.6)	-	-
Ruck	7 (8%)	2.4 (0.6–4.2)	1 (4%)	0.3 (0–1.0)
*Other/unknown*	*11 (12%)*	*3.8 (1.5–6.0)*	*6 (22%)*^∧^	*2.1 (0.4–3.7)*

*Includes jumped, running, slipped, changing direction, etc. ^∧^Includes four unknowns, one maul and one non-specific contact event.

**Table 4:** Exposure time, number, proportion, IR (95%CI), and match injury rate of overall (*n* = 89) and concussion (*n* = 27) injuries, by leagues.

	Proportion of overall time-loss match injuries	Overall time-loss match injury rate (IR) per 1,000 h (95%CI)	Proportion of concussion match injuries	Concussion match injury rate (IR) per 1,000 h (95%CI)
1st league	40%	29.2 (19.5–38.8)	44%	10.0 (4.3–15.7)
2nd league	34%	50.0 (32.1–67.9)	30%	13.3 (4.1–22.6)
3rd league	10%	17.6 (6.1–29.2)	7%	3.9 (0–9.4)
4th league	15%	21.7 (9.9–33.4)	19%	8.3 (1.0–15.6)

## Discussion

The main findings from this study of a 10-week long tournament in 807 amateur student Rugby players were a match-related time-loss injury rate of 30.6 per 1,000 match hours (95% CIs: 24.2–36.9) or 11% of the total cohort. This time-loss match injury rate is significantly less than a meta-analysis of senior male professional Rugby players (Williams et al., 2013) that reported an injury rate of 81 per 1,000 match hours (95% CIs: 63–105), but is comparable with meta-analysis in senior amateur males (46.8/1,000 player hours; 95% CIs: 34.4–59.2; Yeomans et al., 2018) and a pooled analysis of youth (7–21 years old) rugby union players (26.7 per 1,000 h, 95% CIs:13.2–54.1; Freitag et al., 2015). The most comparable specific cohort to the present one is another student rugby population from the United States, which reported a significantly lower match injury rate of 16.9 per 1,000 match hours (95% CI: 15.1–18.9) in males (Kerr et al., 2008). The significantly higher rate in the present cohort could be related to the present cohort being a far wider range of recreational rugby ability, in four different leagues of ability (Kerr et al., 2008).

Of the 89 match-related time-loss injuries reported in the current study, almost half (44%) were to the head region. Besides the head, other commonly injured regions in this cohort were the shoulder (18%), ankle (12%), knee (9%) and hand (6%). Thus, the head and shoulder and ankle together accounted for almost 34 of all injuries. This injury location profile is similar to which was described in this same koshuis cohort in 2003–2005 (Puren et al., 2007), a North American student rugby cohort from 2005 to 2006 (Kerr et al., 2008) and a UK non-professional cohort from 2009 to 2012 (which only reported injuries that resulted in more than 7 days’ time-loss; Roberts et al., 2013). However, the meta-analysis of six studies on amateur Rugby level only reported on the knee, shoulder, thigh, ankle, neck and back, but not head region as a commonly injured areas (Yeomans et al., 2018). Moreover, the rates of head/neck injury in this cohort (14.4; 95% CI: 10.1–18.8) are more similar to the rate reported from professional Super Rugby tournaments between 2012 and 2016 (15.7; 95% CI: 13.1–18.7; Schwellnus et al., 2019). One possible reason for this seemingly elevated rate of head injuries could be World Rugby’s “recognize and remove” policy at the non-professional level^2^, which has effectively lowered the threshold for reporting of concussions whereby suspected concussions need to be removed and are considered as diagnosed concussed unless a medical practitioner declares otherwise. Moreover, awareness, and thus reporting, of concussion could have improved as a result of fear of long-term neurodegeneration from concussions from empirical (Gardner et al., 2014) and popular media (e.g., “Concussion” movie) and class action law suits by former collisions athletes against national governing bodies for neurodegenerative issues. These fairly recent concussion awareness improvements could mean that older studies might have lower rates of head injury as a result of under-reported concussions. For example, one of the studies included in the amateur meta-analysis includes data from as long ago as 1993 (Yeomans et al., 2018).

Indeed, concussion was the most common overall diagnosis in this cohort at a rate of 9.3 per 1,000 match hours (95% CIs: 5.8–12.8). This rate is substantially higher for a study of US college rugby in 2005/6 (2.2 per 1,000 match hours; 95% CIs: 1.5–2.8; Kerr et al., 2008), a Stellenbosch University koshuis cohort from 2012/13 (1.4 per 100 match hours; 95% CIs 1.2–1.6; Mathewson and Grobbelaar, 2015), and a UK community rugby cohort (1.6 per 1,000 match hours; 95%CI: 1.2–1.9; Roberts et al., 2017). Although this rate point estimate (9.3 per 1,000 match hours) is about double that reported by a meta-analysis (Gardner et al., 2014), it falls within the range of point estimates reported (0.19–17.1 per 1,000 h). The difference in rates between the present cohort and the cohorts of 2005/6 and 2012/3 could be attributed to the greater awareness described in the previous paragraph (Roberts et al., 2017). Moreover, the latter study of koshuis players from 2012/13 only reported tackle-related injuries, which would report most, but not all concussions (Mathewson and Grobbelaar, 2015). By comparison, the present study had a tackle-specific concussion rate of 3.1 per 1,000 h (95% CIs: 1.1–3.1), which is no longer significantly higher than the 2012/13 cohort. However, the six-fold higher rate than the more recent six-season study of non-professional Rugby players in the United Kingdom between 2009 and 2015 (IR ratio: 6.4, 95% CIs: 4.3–9.5) is more striking as it would only partly be explained by these differences in awareness, and thus reporting (Roberts et al., 2017). It is unlikely that the higher threshold for injury diagnosis (only those that cause >7 days’ time loss) explains the difference in injury rates with the present cohort as time-loss for most concussions is longer than 7 days, in general. A possible explanation for this difference could be the fact that injury reporting in this UK study relied on the team’s medical staff to report a concussion. In the present cohort, a consistent group of independent first aiders, who were trained in World Rugby concussion guidelines, observed every match looking for any signs/symptoms of suspected concussions. Thus, it is unlikely that any concussions were missed in this study—indeed, it is more likely that concussions were over-reported. However, this over-reporting is a reality at this non-professional level of the game, with an appropriately conservative “recognize and remove” protocol in place that requires all suspected concussions to be removed from the field immediately. Moreover, the UK study also included semi-professional players. The present study’s cohort would be more accurately described as recreational, with a few aspirational semi-professional players. In this way, the present study’s cohort was more homogeneous than that of the UK’s. It should be noted that the present study is a single-season study with 2,915 match hours of exposure while the UK was a six-season study with 1,75,940 match hours (Roberts et al., 2017). It should also be noted that this cohorts’ concussion rate is still substantially lower than has been described in amateur rugby league players in a meta-analysis performed on studies published between 1990 and 2005 (19.1 per 1,000 match hours, 95% CIs: 14.5–25.3; King et al., 2017). The higher rates of concussion in rugby league could be related to differences in the game styles of union and league: league has de-emphasized the scrum and ruck phases of play, meaning that the majority of the game is comprised of tackling, and tackling is highly associated with concussion injuries in rugby league (Gardner et al., 2015; King et al., 2017).

Despite the possibility of over-reporting of concussions in the present study, this high rate of injury warrants further investigation in this cohort. The most frequently reported phase of play associated with concussions was tackling and accidental collisions (not part of tackles). While tackling is commonly associated with concussion in Rugby (Gardner et al., 2014; Roberts et al., 2017), it is unusual that the second-most frequent phase was accidental collisions not part of a formal tackle. When considering contact events (using video surveillance), Roberts et al. (2017) found that collision tackles had a higher propensity for concussions than legal tackles. However, the high proportion of concussions in the present study that were attributed to accidental collisions warrants further investigation. Indeed, the addition of video surveillance to this injury surveillance in future could assist in identifying and confirming these mechanisms associated with injury in the present study. Another interesting finding of this current study was that the overall injury rate was the highest in the second league. Anecdotally, the medical staff felt this could be explained by this league having the largest mismatches in conditioning: while some players in this league are aspirational first league players, some are just wanting to play for fun. The latter proportion could also be increased during high rates of injury that would require third league players to be moved up into these higher leagues. However, this finding did not translate into concussions rates—all leagues had similar concussion rates.

It is important to reiterate the potential limitation of this study. As this is only a one-season study it is difficult to know if this injury rate is a fair reflection, but ongoing surveillance in this cohort will be able to answer this. Also, the total number of matches (*n* = 101) was about half the normal number of matches due to a severe drought in the Western Cape in 2017 and 2018 that affected field availability in the entire region. We are also not able to verify if the 807 reported players all played in the league, but this is likely given that registering incurs a fee. There was also no information on the individual matches that the 807 players played in, so that the IRs can only be considered as crude estimates without adjustment for correlations due to the same players playing in many matches. We are also not able to report which leagues these players played in as this is not known at the time of registration, and is also dynamic throughout the season depending on injuries and form. As a result of these shortcomings, we were left to estimate exposure from the known number of matches and duration of each, assuming 30 players were present for each match, as is suggested by the consensus statement (Gardner et al., 2014). Finally, due to the South African private health care system that functions as a free market on a “fee-for-service” model, it is possible that some non-concussion injuries were missed if a player opted to seek attention outside of the university medical services, which would result in this study, being an under-estimation of the true rate.

In conclusion, this study found a high rate of concussion injuries in these amateur student Rugby players, especially in comparison to this cohort’s relatively low overall match injury rate (Yeomans et al., 2018). However this elevated rate could also be a positive reflection of the heightened vigilance of the medical staff and, in particular, specifically trained first aiders at these matches. Nonetheless, these findings warrant the continued surveillance in order to monitor and, ultimately reduce, these injuries. To this end, it is important to continue to investigate and improve on the effectiveness of concussion education programmes (Fraas and Burchiel, 2016). Moreover, the addition of video surveillance to these matches may provide insight into factors associated with concussions in order to develop a targeted intervention for this cohort.

## Data Availability Statement

The datasets analyzed for this study can be found in the Figshare repository: https://doi.org/10.6084/m9.figshare.8299298.

## Ethics Statement

The studies involving human participants were reviewed and approved by Stellenbosch Health Research Ethics Committee. The patients/participants provided their written informed consent to participate in this study.

## Author Contributions

All listed authors contributed to the conceptualization. JB and LS collected the data and drafted the first version of the manuscript. JB, LS and KS analyzed the data. EJ performed an independent analysis of the data for corroboration. PV and ED reviewed the preliminary data. All authors provided substantial edits to the manuscript and approved the final version.

## Conflict of Interest

The authors declare that the research was conducted in the absence of any commercial or financial relationships that could be construed as a potential conflict of interest.

## Footnotes

^1^https://playerwelfare.worldrugby.org/concussion

^2^https://www.world.rugby/news/134498?lang=en
